# Tacrolimus Decreases Albuminuria in Patients with IgA Nephropathy and Normal Blood Pressure: A Double-Blind Randomized Controlled Trial of Efficacy of Tacrolimus on IgA Nephropathy

**DOI:** 10.1371/journal.pone.0071545

**Published:** 2013-08-19

**Authors:** Yong-Chul Kim, Ho Jun Chin, Ho Suk Koo, Suhnggwon Kim

**Affiliations:** 1 Department of Internal Medicine, Seoul National University Hospital, Seoul, Korea; 2 Department of Internal Medicine, Seoul National University Bundang Hospital, Seong-Nam, Korea; 3 Department of Internal Medicine, Seoul National University College of Medicine, Seoul, Korea; 4 Renal Institute, Seoul National University Medical Research Center, Seoul, Korea; 5 Department of Internal Medicine, Inje University Seoul Paik Hospital, Seoul, Korea; University of Sao Paulo Medical School, Brazil

## Abstract

**Background:**

Treatment remains uncertain for IgA nephropathy patients with mild to moderate proteinuria, for whom anti-hypertensive medication or the RAS blocker is not applicable due to low blood pressure.

**Trial design:**

A double blinded randomized trial.

**Methods:**

The anti-proteinuric effect of tacrolimus was explored for 40 biopsy-proven mild IgA nephropathies for 16 weeks. We randomly assigned patients either to receive tacrolimus or placebo with stratification by using a renin angiotensin system blocker. The primary outcome was the percentage change of final UACR compared to the baseline value (pcUACR).

**Results:**

The mean value of pcUACR at 12-week and 16-week visits (primary outcome) was decreased more in the Tac group compared to the control group (–52.0±26.4 vs –17.3±29.3%, p = 0.001). At each visit, pcUACR was also decreased more in the Tac group compared to the control group. In the Tac group, the pcUACRs were –60.2±28.2%, –62.2±33.9%, –48.5±29.8%, and –55.5±24.0%, and, in the control group, –6.8±32.2%, –2.5±35.9%, –12.7±34.2%, and –21.9±30.6%, at 4-week, 8-week, 12-week, and 16-week visits, respectively. The pre-defined secondary outcomes were better in the Tac group compared to the control group. The frequency of decrease in pcUACR and percentage change of UPCR (pcUPCR) ≥50% at 16 weeks were 65.0% (13/20) and 55.0% (11/20)in the Tac group, and 25.0% (5/20) and 15.0% (3/20), in the control group, respectively (p = 0.025 for pcUACR and p = 0.019 for pcUPCR). However, tacrolimus wasn't effective with a dose of 0.05 mg/kg/day in patients taking ARB. The adverse events were tolerable.

**Conclusion:**

Tacrolimus effectively reduced proteinuria in IgA nephropathy with normal blood pressure. This suggested that tacrolimus could be an alternative to corticosteroid and RAS blocker for IgA nephropathy patients who cannot endure anti-hypertensive medication.

**Trial Registration:**

Clinicaltrial.gov NCT1224028

## Introduction

IgA nephropathy is the most common glomerulonephritis among patients with renal biopsy in Korea [1], [2] as well as in the other countries [3]. In Korea, the incidence of IgA nephropathy among renal biopsies has been increasing over the last 20 years [1], and the estimated cumulative incidence of end stage renal disease (ESRD) is 32.8% for 15 years after renal biopsy [2], which does not significantly differ from other reports in Western countries [4].

The most important clinical parameters to determine the prognosis in IgA nephropathy are proteinuria, hypertension, and glomerular filtration rate (GFR) [5]. Proteinuria was a more important risk factor compared to GFR, represented by serum creatinine in normotensive IgA nephropathy [6]. Many researchers reported the meaningful cutoff criteria of proteinuria was 1 g/day [5], [7]–[11] as the risk for ESRD, but another report suggested proteinuria >0.5 g/day increased the risk [12]. Furthermore, several reports suggested early or mild IgA nephropathy with minimal or no proteinuria was not benign, especially in Asians [13], [14]. A report from a study carried out in China stated that, among IgA nephropathy patients with proteinuria of <0.4 g/day, GFR ≥90 ml/min/1.73 m^2^, and normotension, proteinuria increased in 46% of patients, hypertension was developed in 38%, and renal insufficiency in 24% during the mean follow-up duration of 111 months [13]. In another report from a study in Hong Kong, proteinuria of >1 g/day developed in 33% of patients, hypertension in 26%, and renal insufficiency (GFR <70 ml/min/1.73 m^2^) in 7% during the median follow-up of 7 years in IgA nephropathy patients with proteinuria of <0.4 g/day, normal renal function, and normotension [14].

An optimized supportive therapy is the key strategy for IgA nephropathy patients at risk of progression [5], in which the renin-angiotensin-system (RAS) blocker is the most important non-immunosuppressive treatment [5]. However, treatment is uncertain for IgA nephropathy patients with mild to moderate proteinuria, for whom anti-hypertensive medication or the RAS blocker is not applicable because of low blood pressure. While the currently suggested therapy for the proteinuric patients, despite receiving optimized supportive care, is corticosteroid, most studies included patients with moderate to severe proteinuria who were being administered RAS blocker, and/or hypertension [15], [16] and it is not clear whether corticosteroid therapy would effectively compensate for the adverse events to prevent renal deterioration in patients with mild to moderate proteinuria and normal blood pressure.

Recently, Zhang et al. improved proteinuria of 14 refractory IgA nephropathy patients who were receiving tacrolimus and moderate doses of prednisolone [17]. They showed that the expression of synaptopodin and calcinueurin in renal tissue from the patients was partially normalized after treatment, which was reported as the non-immunological effects of tacrolimus [18], and suggested tacrolimus could improve proteinuria without serious adverse events in IgA nephropathy [17].

Therefore, we tried to verify the anti-proteinuric effect of tacrolimus for IgA nephropathy patients with normotension or normal blood pressure with a RAS blocker, normal renal function, and mild to moderate proteinuria, who were not able to tolerate additional anti-hypertensive or RAS blockers for a short-term period.

## Materials and Methods

### Trial design

The protocol for this trial and supporting CONSORT checklist are available as supporting information: see Checklist S1 and Protocol S1.his study was a double blind randomized controlled clinical trial and was performed in a single center (clinicaltrial.gov identifier: NCT01224028). The protocol was approved by the Institutional Review Board of Seoul National Universtiy Hospital (IRB number: H-1002-032-309). There was no change of methods after trial commencement to declare. After obtaining written informed consent from all participants, we randomized patients 1:1 to a control group (placebo) or to a Tac group who had received tacrolimus, in a double blind manner and stratified according to using a RAS blocker, using the computer-generated randomization lists by the independent statistical committee from the researcher (doctors, nurses, and pharmacists related to this study) and patients. We followed the patients at 1 week, 4 weeks, 8 weeks, 12 weeks, and 16 weeks after a baseline visit for randomization.

### Participants

The inclusion criteria were a biopsy proven IgA nephropathy, aged ≥18 and <70 years, serum creatinine ≤1.5 mg/dL or estimated GFR ≥45 ml/min/1.73 m^2^, urine albumin to creatinine ratio (UACR) ≥0.3 and <3.0 g/g creatinine, and blood pressure (BP) less than 130/80 mmHg during the 3-month period before randomization. The GFR was estimated by the equation of the 2009 CKD-EPI creatinine equation [19]. We excluded the patients with ≥20% variations of BPs, UACRs, serum creatinines during 3 months before randomization, or with potassium sparing diuretics, corticosteroid, immunosuppressive medication, omega-3 fatty acid, or two or more medications of renin angiotensin system blocker (RAS blocker). We permitted the use of one RAS blocker drug, although we did not allow any change of medication and dose of medication during 3-month period before and after randomization. Other exclusion criteria were pregnancy or secondary IgA nephropathy.

### Interventions

The initial dose of tacrolimus was 0.1 mg/kg/day administered orally in two divided doses and was titrated to maintain trough levels at 5–10 ng/ml at each visit after randomization. If the level was ≥15 ng/ml, we stopped tacrolimus for 2 weeks and then re-measured the trough level to adjust the dosage as described above. The level of tacrolimus was not given to the patient or the researcher but only to the statitistical committee member in charge of this study who decided on the dose of tacrolimus or placebo and notified such to the pharmacist at each visit before prescription. For adjusting the dose of placebo, the committee member should change the number of placebo capsules for the patient in the control group in the same manner as the adjustment of medication for the patient with tacrolimus who visited on the most recent day with a random allocation (no change, increase or decrease of dose, or discontinue of prescription). After 8 weeks of randomization, we reduced the dose of tacrolimus to 0.05 mg/kg/day or to half of the decided dose to maintain the trough level in 5–10 ng/ml at the 8-week visit and continued this up to 16 weeks after randomization.

### Outcomes

We defined the baseline value of UACR or UPCR as the mean value of UACR or UPCR during the 3-month screening period and at visit 1 for randomization. The final level of UACR was defined as the mean value of UACR at 12-week and 16-week visits. The primary outcome was defined as the percent change (%) of final UACR (pcUACR) compared to the baseline value [100 x (final UACR- baseline UACR)/baseline UACR]. We defined several secondary outcomes related to the percentage changes of UACR and UPCR (pcUPCR). There was no changes of outcome-criteria.

### Sample size

We estimated the sample size based on previous studies [20]–[24] that showed the mean pcUACR 35.4% (standard deviation, 36.7%). For comparison of the control and Tac groups at a level of significance of 5% (α-error  = 0.05), we calculated that at least 17 patients were needed in each group to have 80% percent power (ß-error  = 0.20). We allocated 20 patients to each group with an estimated ≤15% drop-out rate.

### Statistical analysis

Analysis of primary and secondary outcomes was by intention-to-treat (ITT). For statistical analysis, we used a paired or unpaired Student *t-*test for continuous variables and Pearson's Chi-square test or Fisher's exact test for qualitative variables. The data throughout the follow-up period were analyzed with the ANOVA for repeated measurements. When we compared parameters between Tac and control groups in each follow-up period during 5 visits, we used the Bonferroni correction for the criterion of significant difference between groups as p-value <0.01 (0.05/5).

## Results

**Figure 1:**
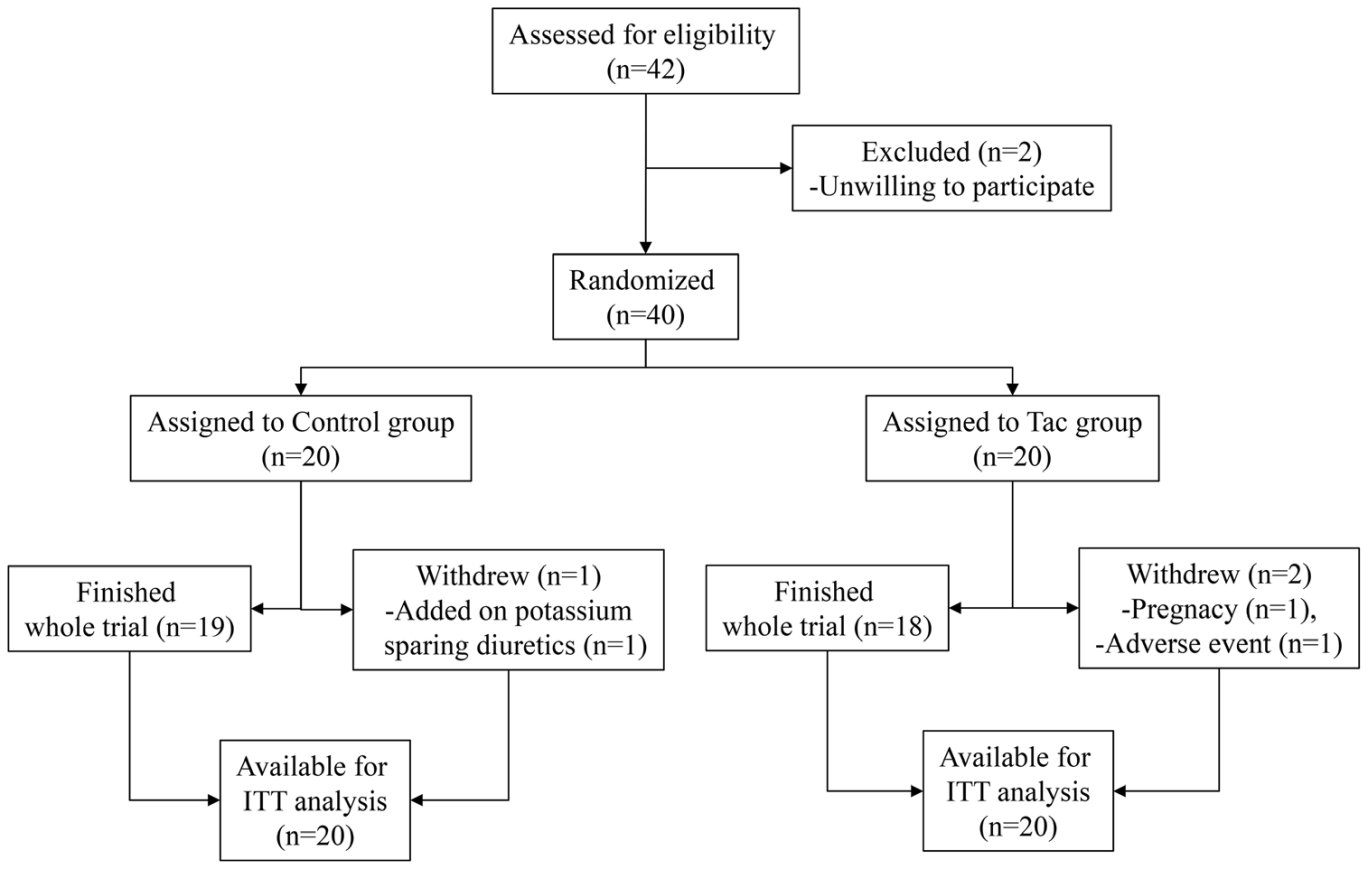
Study algorithm. One patient in the control group withdrew at the 8-week visit because of the addition of a prohibited drug in another department, one patient in the Tac group withdrew at day 1 after enrollment because of pregnancy and had taken only 2 mg of tacrolimus, and another patient in the Tac group withdrew at the 4-week visit because of general weakness and myalgia related to medication.

The patients were enrolled from 26^th^ Nov. 2010 to 15^th^ Feb. 2011. Among 42 eligible patients with IgA nephropathy, 40 patients were allocated to control group (20 patients including 9 using a RAS blocker) or Tac group (20 patients including 11 using a RAS blocker) with stratification according to using RAS blocker except 2 patients unwilling to participate in this study (Figure 1). Kidney biopsy was performed 45.2±57.8 months before the study (ranging from 0 to 243 months). The RAS blockers which patients had been taking were angiotensin II type I receptor blockers (ARBs); eleven patients with valsartan (one patient with 40 mg/day and ten patients with 80 mg/day), eight patients with candesartan (one patient with 4 mg/day and seven patients with 8 mg/day), and one patient with losartan (50 mg/day). In the control group, one patient was excluded because they had taken potassium sparing diuretics in the other clinic at an 8-week visit, while in the Tac group, one patient declined the consent because of general weakness and myalgia at a 4-week visit and the other patient was withdrawn at day 1 after enrollment because of a positive result for pregnancy screening. She was unaware of her pregnant status and had taken 2 mg of tacrolimus. The compliance to take placebo or tacrolimus was 91.3%±10.3% in the control group and 91.5%±7.4% in the Tac group and did not differ between groups.

### Baseline characteristics

All parameters did not differ between the control and Tac groups at enrollment (Table 1). Among patients without a RAS blocker, only DBP in the Tac group was different compared to the control group (72±3 vs 76±4 mmHg, p = 0.029). Among patients with a RAS blocker, clinical characteristics did not differ between groups (all p>0.1).

**Table 1:** The basal characteristics of patients.

		Control (20)	Tac (20)	p-value
At screening visits during 3 months before randomization	Hypertension (%)	7 (35.0)	9 (45.0)	0.748
	Diabetets Mellitus (%)	0 (0.0)	1 (5.0)	1.000
	DBP (mmHg)*	73±5	73±5	0.775
	SBP (mmHg)*	119±6	118±7	0.603
	Creatinine (mg/dL)	0.98±0.24	1.05±0.29	0.452
	GFR (ml/min/1.73 m^2^)	83.3±22.4	80.1±21.5	0.645
	UACR (mg/g cr)*	910±561	975±450	0.692
	UPCR (mg/g cr)*	1193±664	1291±535	0.609
At enrollment visit	Age (years)	40.1±12.8	36.9±11.4	0.403
	Sex (Male, %)	6 (20.0)	6 (20.0)	1.000
	RAS blocker (%)*	9 (45.0)	11 (55.0)	0.527
	Duration of illness (months)*	49.4±59.8	41.2±57.1	0.661
	BMI (kg/m^2^)	23.3±4.5	22.5±3.8	0.574
	DBP (mmHg)	74±6	73±3	0.775
	SBP (mmHg)	120±7	117±8	0.603
	Protein (g/L)	7.1±0.4	7.1±0.5	0.621
	Albumin (g/L)	4.1±0.3	4.1±0.3	0.860
	Cholesterol (mg/dL)	184±30	190±38	0.576
	LDL-Cholesterol (mg/dL)	110±27	103±33	0.513
	Hemoglobin (g/dL)	13.4±1.5	13.5±1.8	0.743
	C-reactive protein (mg/dL)	0.098±0.145	0.044±0.062	0.134
	Creatinine (mg/dL)	0.98±0.26	1.06±0.30	0.379
	GFR (ml/min/1.73 m^2^)*	84.6±23.2	79.6±21.6	0.482
	45–59 (%)	4 (20.0)	4 (20.0)	
	60–89 (%)	6 (30.0)	8 (40.0)	
	≥90 (%)	10 (50.0)	8 (40.0)	
	UACR (mg/g cr)*	965±459	1098±635	0.452
	UPCR (mg/g cr)*	1202±500	1398±809	0.362
	300–999 (%)	8 (40.0)	8 (40.0)	
	1000–1999 (%)	11 (55.0)	9 (45.0)	
	2000–2999 (%)	1 (5.0)	3 (15.0)	
	Hematuria*	15 (75.0)	13 (65.0)	0.490
Pathologic findings by Oxford classification*	M score (1, %)	11 (64.7)	5 (31.3)	0.055
	S score (1, %)	12 (70.6)	14 (87.5)	0.235
	E score (1, %)	2 (11.8)	6 (37.5)	0.118
	T score			0.607
	0	14 (82.4)	11 (68.8)	
	1	2 (11.8)	4 (25.0)	
	2	1 (5.9)	1 (6.3)	

* Tac: patients with tacrolimus. DBP: diastolic blood pressure. SBP: systolic blood pressure. GFR: calculated with 2009 CKD-EPI creatinine equation. UACR: urine albumin to creatinine ratio in mg/g creatinine unit. UPCR: urine protein to creatinine ratio in mg/g creatinine unit. RAS blocker: renin angiotensin system blocker using angiotensin II type I receptor blocker (ARB). Duration of illness: time-duration from renal biopsy to this clinical trial. BMI: body mass index. Hematuria: RBC 5 or more examined in 400-fold fields by light microscopic examination. Pathologic findings by Oxford classification: retrospectively reclassified findings in 33 patients using Oxford classification of IgA nephrothy.

We re-analyzed the pathologic findings of 33 patients (82.5%) with Oxford classification of IgA nephropathy [25]. The frequency of patients with M1 score was slightly higher in control group (11/17 in control group vs 5/16 in Tac group) but was not significant (p = 0.055). Other findings including frequencies of S score (0, 1), E score (0, 1), and T score (0, 1, 2) were not different between groups (Table 1).

### Primary outcome

**Figure 2:**
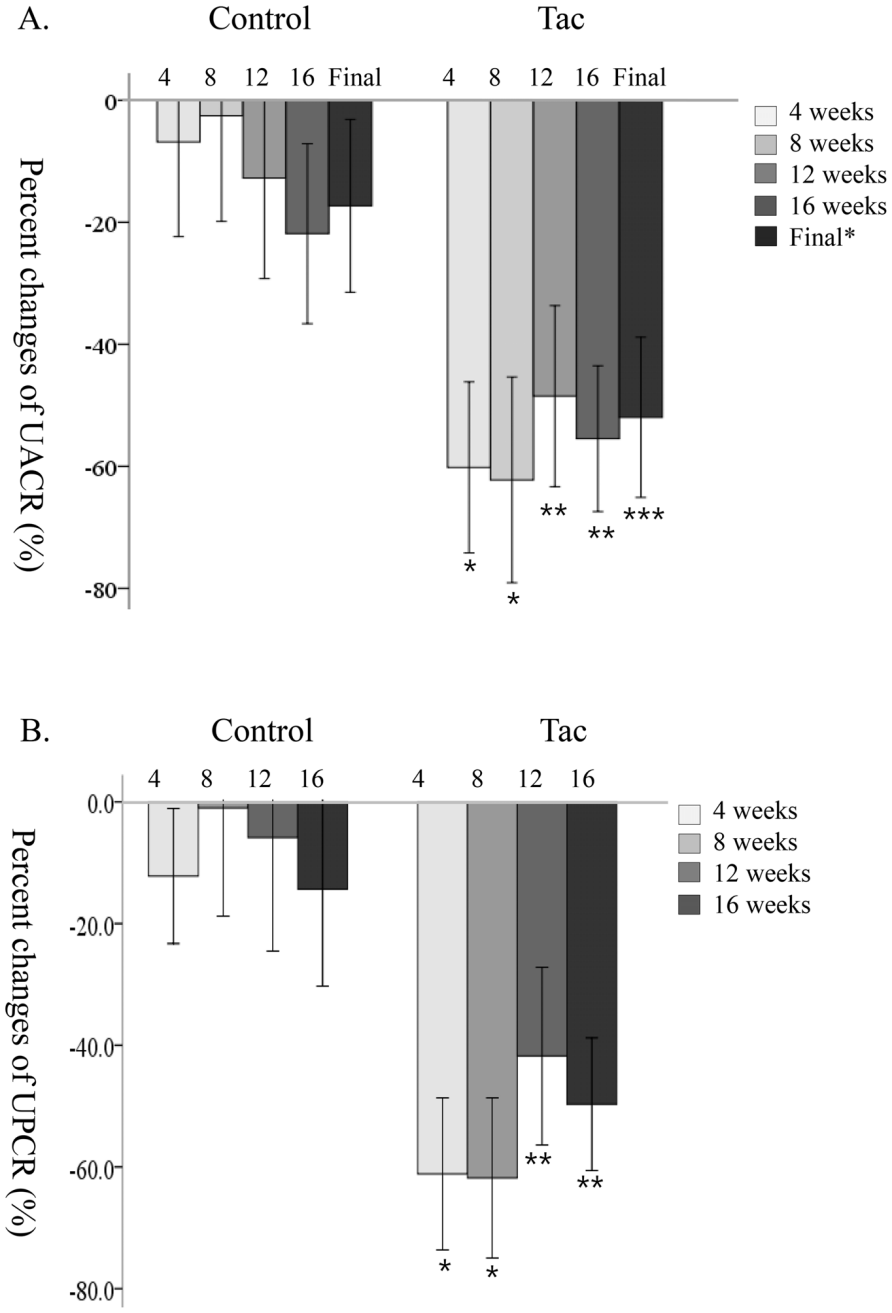
The percentage changes of UACR and UPCR at each visit compared to the baseline level. 2A. Percent changes of UACR. 2B. Percent changes of UPCR. Baseline UACR or UPCR; mean value of UACR or UPCR at screening period and randomization. Final; mean value of UACR at 12 weeks and 16 weeks. The bar in each bar graph is the 95% confidence interval of mean value of the percent change of UACR or UPCR at each visit compared to baseline level. * p-value <0.001, **p<0.01, *** p = 0.001 by Student *t*-test for percent change of UACR or UPCR between control and Tac groups at each visit.

The pcUACR at each visit compared to at baseline was calculated. The primary outcome, defined as the mean value of pcUACR at 12-week and 16-week visits, was decreased more in the Tac group compared to the control group (–52.0±26.4 vs –17.3±29.3%, p = 0.001). At each visit, pcUACR was also decreased more in the Tac group compared to the control group (Figure 2A). In the Tac group, the pcUACRs were –60.2±28.2%, –62.2±33.9%, –48.5±29.8%, and –55.5±24.0% at 4-week, 8-week, 12-week, and 16-week visits, respectively. The decreased amount of pcUACR at a 12-week visit, which was 4 weeks after decreasing the dose of tacrolimus from 0.1 mg/kg/day to 0.05 mg/kg/day, was lower than that of the 4-week visit (p = 0.030 by repeated measured ANOVA). However, the pcUACR at the 16-week visit did not differ from the pcUACR at the 4-week visit or at the 8-week visit (p>0.05 by repeated measured ANOVA) in the Tac group. In the control group, pcUACRs were –6.8±32.2%, –2.5±35.9%, –12.7±34.2%, and –21.9±30.6% at 4-week, 8-week, 12-week, and 16-week visits, respectively. The decreased amount of pcUACR at the 16-week visit was greater than that of the 4-week visit in the control group (p = 0.032 by repeated measured ANOVA).

### Secondary outcome

In the Tac group, the pcUPCRs were –61.1±25.2%, –61.8±26.4%, –41.8±29.4%, and –49.7±21.9% at 4-week, 8-week, 12-week, and 16-week visits, respectively. The decreased amount of pcUPCRs at the 12-week visit was lower than that at the 4-week or 8-week visits (p = 0.003 and p = 0.031 by repeated measured ANOVA, respectively) and the decreased amount of pcUPCRs at the 16-week visit was lower than that at the 4-week visit in the Tac group (p = 0.022 by repeated measured ANOVA). In the control group, pcUPCRs were –12.2±23.1%, –0.98±36.9%, –5.8±37.2%, and –14.4±40.0% at 4-week, 8-week, 12-week, and 16-week visits, respectively (Figure 2B). The pcUPCRs at each visit did not differ in the control group (p>0.05 by repeated measured ANOVA, respectively). The pcUPCR at the 16-week visit in the Tac group decreased more than the control group (p = 0.004) and at each visit, pcUPCR decreased more in the Tac group compared to the control group (Figure 2B).

**Figure 3:**
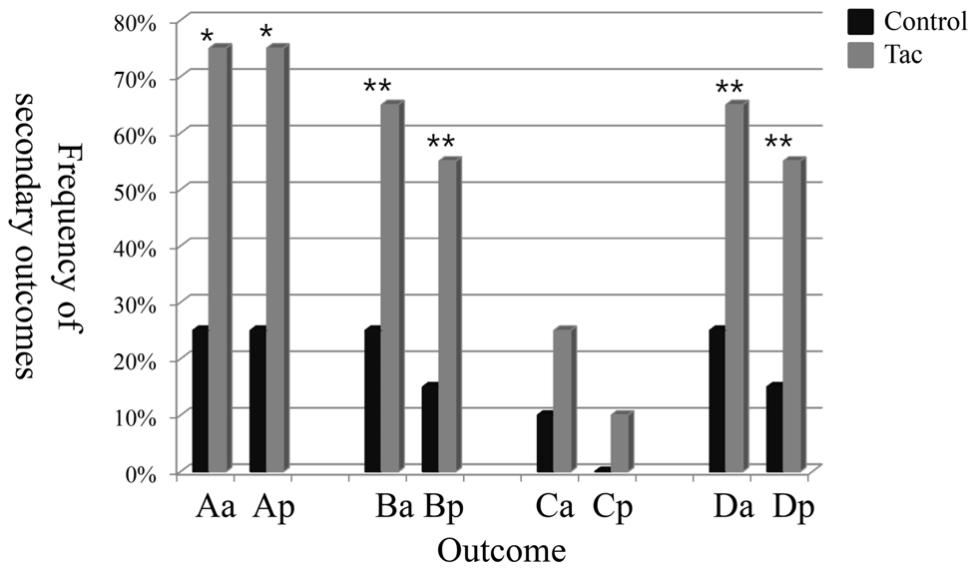
The frequency of decrease in UACR and UPCR at 16 weeks as secondary outcomes. Outcome Aa and Ap: frequency of decrease in UACR and UPCR ≥30% at 16 weeks, compared to baseline level. Outcome Ba and Bp: frequency of decrease in UACR and UPCR ≥50% at 16 weeks compared to baseline level, Outcome Ca and Cp: frequency of decrease in UACR and UPCR <200 mg/g cr at 16 weeks. Outcome Da and Dp: frequency of outcome B and/or C. *p<0.01, **p<0.05; difference of frequency between groups.

The pre-defined secondary outcomes were better in the Tac group than in the control group (Fig. 3). The frequencies of decrease in pcUACR and pcUPCR ≥50% at 16 weeks were 65.0% (13/20) and 55.0% (11/20) in the Tac group, and 25.0% (5/20) and 15.0% (3/20) in the control group (p = 0.025 for pcUACR and p = 0.019 for pcUPCR). The proportion of patients with UACR <0.2 g/g cr at the 16-week visit tended to be greater in the Tac group (25.0%) compared to the control group (10.0%), although it did not show statistical significance.

### Repeated measurements

Blood pressures between groups did not differ throughout the study period. The serum creatinine levels were higher at 4-week and 8-week visits in the Tac group than in the control group, although the p-values did not show significant differences with the criterion by Bonferroni's correction. After the 8-week visit, the levels of creatinine did not differ between groups. The levels of UACR and UPCR were lower in the Tac group at 4-week and 8-week visits than in the control group. The serum trough level of tacrolimus was maintained within 5–10 ng/ml up to 8 weeks and then decreased along with a reduction of dosage (Table 2).

**Table 2:** The change of absolute values of blood pressures, serum creatinine, GFR, UACR, and UPCR during follow-up period.

		V1 (0 week)	V3 (4 weeks)	V4 (8 weeks)	V5 (12 weeks)	V6 (16 weeks)	*p-value* *
DBP	Control	74±6	77±9	74±8	77±7	73±9	*0.635*
(mmHg)	Tac	72±3	75±7	76±6	74±7	75±5	
	*p-value* **	*0.138*	*0.438*	*0.347*	*0.250*	*0.747*	
SBP	Control	120±7	121±25	123±10	122±8	120±11	*0.925*
(mmHg)	Tac	117±8	122±11	122±11	124±6	122±9	
	*p-value* **	*0.192*	*0.911*	*0.860*	*0.512*	*0.381*	
Creatinine	Control	0.98±0.26	0.97±0.27	0.96±0.28	1.02±0.29	0.99±0.27	*0.261*
(mg/dL)	Tac	1.06±0.30	1.17±0.32	1.18±0.33	1.13±0.36	1.10±0.32	
	*p-value* **	*0.355*	***0.048***	***0.033***	*0.332*	*0.291*	
GFR	Control	84.6±23.2	84.4±24.7	85.6±24.1	80.2±25.4	83.1±24.1	*0.143*
(ml/min/1.73 m^2^)	Tac	79.6±21.6	71.5±21.4	70.4±21.0	75.8±23.0	77.4±22.9	
	*p-value* **	*0.637*	*0.101*	***0.049***	*0.582*	*0.472*	
UACR	Control	965±459	841±415	898±524	779±426	700±386	***0.021***
(mg/g cr)	Tac	1098±635	405±383	343±284	538±493	601±565	
	*p-value* **	*0.943*	***0.002***	***<0.001***	*0.120*	*0.071*	
UPCR	Control	1202±500	1061±531	1191±707	1071±497	973±471	***0.009***
(mg/g cr)	Tac	1398±809	501±425	458±317	761±590	863±798	
	*p-value* **	*0.912*	***0.001***	***<0.001***	*0.092*	***0.033***	
Trough level oftacrolimus (ng/ml)	Tac	-	6.56±2.95^#^	6.42±3.53^#^	4.64±4.17	3.09±1.87	***0.001***

* p-value between control and Tac group by repeated measured ANOVA during follow-up period.

#p-value <0.001 comapred to trough level of tacrolimus at visit 6 by posthoc analysis in a repeated measured ANOVA.

** p-value by Student t-test: The significant difference was considered as p-value <0.01 by Bonferroni correction.

The dose of tacrolimus was decreased from 0.05 mg/kg bid per day to 0.025 mg/kg bid per day after 8 week-visit.

### Outcomes according to ARB

We compared the outcomes between groups according to ARB use at randomization (Table 3). The results of pcUACR and pcUPCR at each visit were better in the Tac group than in the control group in patients without ARB medication, even with the p-value criterion of <0.01 by Bonferroni's correction. Among patients with ARB, the pcUACR and the pcUPCR decreased more in the Tac group than in the control group up to the 8-week visit and the difference was not apparent after the reduction of the tacrolimus dose, although the decreased amount of pcUACR or pcUPCR tended to be higher in the Tac group.

**Table 3:** The clinical results of patients stratified with ARB medication at randomization.

	Patients without ARB*			Patients with ARB*		
	Control (n = 11)	Tac (n = 8)	p-value	Control (n = 8)	Tac (n = 10)	p-value
Percent changes of UACR *						
At 4 weeks	–10.9±21.5	–74.4±18.3	***<0.001***	–1.2±44.4	–48.8±30.3	***0.015***
At 8 weeks	–12.8±26.2	–70.5±32.8	***0.001***	11.6±44.0	–55.6±35.0	***0.002***
At 12 weeks	–14.5±31.6	–64.7±15.7	***0.001***	–10.4±39.6	–35.5±32.7	0.160
At 16 weeks	–13.6 ± 27.8	–60.0±31.9	***0.001***	–29.4±32.5	–41.3±25.8	0.372
Final value*	–14.0±26.6	–67.4±12.4	***0.003***	–21.8±34.2	–39.6±28.6	0.246
Percent changes of UPCR						
At 4 weeks	–12.9±17.1	–75.0±16.7	***<0.001***	–11.2±30.8	–50.1±26.0	***0.010***
At 8 weeks	–7.3±33.4	–68.7±28.3	***0.001***	7.7±41.8	–56.3±24.9	***0.003***
At 12 weeks	–6.3±35.5	–58.1±17.9	***0.002***	–5.2±45.3	–28.7±30.9	0.209
At 16 weeks	–6.8±30.8	–52.3±30.3	***0.004***	–21.7±34.1	–36.1±26.3	0.301
% of secondary outcomes at 16 weeks by Fisher's exact test*						
% of UACR decreased ≥30%	18.2	88.9	***0.005***	33.3	63.6	0.370
% of UACR decreased ≥50%	18.2	88.9	***0.005***	33.3	45.5	0.670
% of UACR <200 mg/g cr	0.0	33.3	0.074	22.2	18.2	1.000
% of UPCR decreased ≥30%	18.2	88.9	***0.005***	33.3	63.6	0.370
% of UPCR decreased ≥50%	18.2	77.8	***0.022***	11.1	36.4	0.319
% of UPCR <200 mg/g cr	0.0	11.1	0.450	11.1	36.4	0.319

* ARB: Angiotensin II type I receptor blocker. Percent changes of UACR: calculated by level of UACR at each visit compared to level of baseline. Final value: mean value of UACR at 12 weeks and 16 weeks. Secondary outcome: levels of UACR or UPCR at 16 weeks compared to level of baseline. Baseline value: mean value of UACR or UPCR at screening period and randomization period (0 week).

### Adverse events

The frequency of adverse events was higher in the Tac group, although the severity of most events (43/49) was mild. The frequency of adverse events related to medication tended to be higher in the Tac group, but this was not significant (16/49 events in the Tac group vs 1/15 events in the control group). The symptoms related to tacrolimus were gastrointestinal discomforts, headache, tremor, and coldness of extremities. Only one patient should discontinue the tacrolimus because of general weakness and myalgia after 4 week-medication. Newly onset diabetes mellitus (DM) among patients without DM at randomization, was observed in one patient in the tacrolimus group at the 16-week visit [fasting glucose (96 mg/dL) and HbA1c (6.7%)] (Table 4).

**Table 4:** Adverse reactions during study.

Symptom	Control	Tac
Number of events	15	49
Cardiovascular	1	2
Gastrointestinal	4	21
Genitourinary	0	4
Hematologic	0	1
Musculoskeletal	3	3
Neurologic	1	12
Respiratory	5	4
Dermatologic	1	2
Severity		
Mild	15	43
Moderate	0	6
Severe	0	0
Related to medication	1	16
Cessation of mediation	0	1

## Discussion

We performed a double blinded randomized controlled study to verity anti-proteinuric effect of tacrolimus for IgA nephropathy patients with normotension or normal blood pressure, and mild to moderate proteinuria. Tacrolimus was effectively decreased proteinuria during 16 weeks, compared to placebo. The anti-proteinuric effect of tacrolimus was an additive to a RAS blocker and was dose dependent in patients with a RAS blocker.

We used the placebo as same capsules as the tacrolimus and adjusted the dose of placebo according to the change of tacrolimus dose to achieve complete double blindness for patients and researchers including pharmacists.

Proteinuria is a well-known prognostic factor for ESRD in IgA nephropathy. Traditionally, the prognostic importance of proteinuria was analyzed in gram unit/day [4], [7]–[11]. The proteinuria amount of 1 g/day was the cutoff level to indicate a worse renal prognosis. However, several considerations are involved with this point of view. At first, IgA nephroapthy with proteinuria <1 g/day did not always indicate as benign. In Japan, renal insufficiency was developed during a mean follow-up period of 6.7 years in 17.2% of 203 IgA nephropathy patients with proteinuria 0.5–0.9 g/day and in 3.5% of 197 patients with proteinuria <0.5 g/day [12]. They had a mean creatinine level of 0.95 and 0.84 mg/dL at renal biopsy, respectively. Among 72 Chinese patients with proteinuria <0.4 g/day and creatinine <120 μmol/L, 44% of patients developed proteinuria ≥1 g/day, 26% developed hypertension, and 7% developed renal insufficiency during the median follow-up period of 84 months in Hong-Kong [14]. In main-land China, 46% of patients showed increased proteinuria, 38% developed hypertension, and 24% developed renal insufficiency among 177 IgA nephropathy patients with proteinuria <0.4 g/day and GFR ≥90 ml/min/1.73 m^2^ during the mean follow-up period of 111 months [13]. However, in Caucasians, the long-term outcomes of IgA nephropathy with minimal or no proteinuria was excellent [26]. It is possible that the prognosis of IgA nephropathy among different races differs, partly because of genetic susceptibility [5]. Secondly, as Reich et al. discussed, the course of IgA nephropathy with initial proteinuria <1 g/day is variable according to the change of proteinuria during the follow-up period [27]. The greater increase in proteinuria, the worse the renal survival [27]. In another report, among 121 IgA nephropathy patients with proteinuria ≥1 g/day at presentation or during follow-up period, reduced proteinuria group (in which proteinruia was decreased to <1 g/day at last follow-up) showed better outcome compared to persistent proteinuria group and showed similar renal outcome as in low proteinuria group [7]. Therefore, it is necessary to reconsider the treatment strategy for patients with “low risk proteinuria” and complete or partial remission of proteinuria could be a target treatment to prevent renal progression.

The enrolled patients had proteinuria in spite of appropriate blood pressure with or without anti-hypertensive medication including a RAS blocker. We could not use the full dose of a RAS blocker or combination of RAS blockers because of the relatively low blood pressure. Until now, while no evident guidelines have been available to treat such patients, a high-dose corticosteroid is recommended [5]. Meta-analyses on the effectiveness of corticosteroid for IgA nephropathy showed that steroids provided renal protection but increased the risk of adverse events [15], [16]. In this study, tacrolimus reduced proteinuria in IgA nephropathy patients. The anti-proteinuric effect of tacrolimus was observed as treating several renal diseases including nephrotic syndrome [28], primary glomerulonephritis [29], minimal change lesion [30], membranous nephropathy [31], lupus nephritis [32], [33], and transplanted kidney [34] as well as IgA nephropathy [17]. The possible mechanism of the calcineurin inhibitor to reduce proteinuria is probably multifactorial, and mechanisms other than the immunosuppressive effects may be involved [31]. Zhang et al. demonstrated an increased expression of calcineurin and decreased synaptopodin were recovered after treatment with prednisolone and tacrolimus in renal tissue of IgA nephropathy [17]. They suggested that the anti-proteinuric effect of tacrolimus in IgA nephropathy would result from the stabilization of cytoskeleton in podocytes as the result of Faul's works using cyclosporin [18]. The time required to achieve remission was less than 1 month in 7/9 patients [17]. We also observed that the amount of decrease in UPCR was fully achieved in the short-term period, so the anti-proteinuric effect of tacrolimus was from the non-immunologic mechanism rather than immunosuppressive processes [17]. In this study, GFR was decreased slightly but significantly after 8 weeks medication in the Tac group and, at that time, UPCR was decreased at maximal level. After reduction of the tacrolimus dose, the extent of pcUPCR was reduced, but was still greater in the Tac group than in the control group. This suggested that intraglomerular hemodynamic changes with disturbances of cytokines such as endothelin and prostacyclin in the kidney [35] could be one of the mechanisms to reduce proteinruia by decreasing the permeability to protein as proposed by Chen et al [31].

The anti-proteinuric effect of tacrolimus was also effective for patients taking an ARB and showed usefulness of tacrolimus for add-on therapy after a RAS blocker. Under a RAS blocker, a serum trough level of 5–10 ng/ml was needed to add an anti-proteinuric effect by tacrolimus. For patients who did not take a RAS blocker, the lower dose of tacrolimus was effective to reduce proteinruia. However, the effective dose of tacrolimus to reduce proteinuria was not defined in renal diseases. In a report on a study in China, the authors started with 0.05–0.1 mg/kg/day of tacrolimus and adjusted the dose according to the trough level of 5–10 ng/ml. In other reports, a lower dose (0.05 mg/kg/day) and fixed dose (2–3 mg/day) were also used [31], [32]. The duration of treatment was also variable from study to study. In a study for the treatment of 24 adult patients with steroid-resistant nephrotic syndrome including two IgA nephroapthies, the dose of tacrolimus was maintained to achieve a trough level of 5–10 ng/ml for the initial 6 months and then decreased to a target trough level of 4–6 ng/ml for another 6 months [28]. They achieved significant complete remission of proteinuria during the initial 6 months and there was no rebound of proteinuria during the subsequent 6 months after dose reduction [28].

The adverse events directly related to tacrolimus were tolerable but one patient need to discontinue tacrolimus because of severe subjective general weakness, who had been recovered after cessation of medication, completely. However, the decrease of GFR tended to be with the improvement of proteinuria, implying a hemodynamic mechanism for anti-proteinuric effect by tacrolimus, and there might be concern for the nephrotoxicity by tacrolimus with long-term use. That should be confirmed with other studies to verify pros and cons of tacrolimus on the renal progressoin of IgA nephropathy.

This study was a double blinded randomized study given high level evidence, but had several limitations because of short duration of trial and surrogate marker of proteinuria, not hard outcomes, to be assessed. The treatment with tacrolimus for 16 weeks would not be sufficient to induce a prolonged anti-proteinuric effect. This information suggests a long-term trial is needed with tacrolimus for IgA nephropathy and the appropriate dose of tacrolimus should be determined in further studies.

In conclusion, tacrolimus reduced proteinuria effectively and rapidly in IgA nephropathy with mild to moderate proteinuria and normal blood pressure in this short-term trial with double blinded randomization. This study suggested that tacrolimus could be an alternative to corticosteroid and RAS blocker for IgA nephropathy patients who are not able to tolerate anti-hypertensive medication.

## Supporting Information

Protocol S1 **Protocol for this clinical trial.**
(DOCX)

Checklist S1 **CONSORT Checklist.**
(DOC)
