# Supplementary material for: Tacrolimus Decreases Albuminuria in Patients with IgA Nephropathy and Normal Blood Pressure: A Double-Blind Randomized Controlled Trial of Efficacy of Tacrolimus on IgA Nephropathy
Source: PLoS One. 2013 Aug 19;8(8):e71545. doi: 10.1371/journal.pone.0071545 (PMC3747236; doi:10.1371/journal.pone.0071545)
Supplement: Protocol S1 — Protocol for this clinical trial. (DOCX) [file pone.0071545.s001.docx]

Double-blind, randomized placebo-controlled clinical trial

for the efficacy and safety of

a calcineurin inhibitor, tacrolimus (Prograf cap^®^)

in the patient with non-nephrotic albuminuric, normotensive IgA nephropathy

Study Number: PRGNS-10-01-KOR

Protocol Version: 2.31

Nov 23, 2010

Astellas Pharma Korea, Inc.

Summary

1. Title of clinical trial: Double-blind, randomized placebo-controlled clinical trial for the efficacy and safety of a calcineurin inhibitor, tacrolimus(Prograf cap^®^) in the patient with non-nephrotic albuminuric, normotensive IgA nephropathy

2. Institution : Seoul National University College of Medicine, Seoul National University Hospital

3. Collaborator and Sponsor: Astellas Pharma Korea, Inc. Seoul, Korea

4. Primary Investigator: Prof. Suhnggwon Kim, MD, PhD.

5. Duration of Study: Nov. 2010~ Dec.2012

6. Study Design:

Double blind, placebo-controlled, randomized controlled clinical trial, Phase II

7. Patients group: Patients with IgA nephropathy, biopsy-proven

8. Purpose of the study: This study is to evaluate efficacy and safety of tacrolimus in the patients with non-nephrotic albuminuric, normotensive IgA nephropathy

after 16 week treatment with tacrolimus (Prograf) or placebo.

9. Background: 1) IgA nephropathy is common.

2) The standard conservative therapy for IgA nephroapthy is RAS inhibitor but, low blood pressure is the limitation for using RAS inhibitor.

3) Calcineurin inhibitor is known to decrease proteinruia by inhibition of calcineurin in podocyte and stabilization of cytoskeleton in podocyte in addition to immunosuppressive effect.

4) Proteinuria is the well known surrugate marker for renal progression in glomerulonephritis including IgA nephropathy.

5) This study was designed to verify anti-proteinuric effect of tacrolimus in IgA nephropathypatients with normal blood pressure.

10. Inclusion criteria

1) Patients with IgA nephropathy confirmed by renal biopsy,

2) Serum creatinine measurement ≤1.5mg/ml or MDRD estimated GFR ≥ 45ml/min/1.73m2 (MDRD: Modification of Diet in Renal Disorder),

3) UACR level between 0.3 and 3.0**, and**

4) Blood pressure measurements < 130/80mmHg

11. Exclusion criteria:

1) Use of immunosuppressants for more than two weeks within last one month,

2) Concomitant use of ACE inhibitor, ARB, steroids or immunosuppressant, NDHP-CCB, diuretics, omega-3 fatty acids and its analogue & additional dietary to treat igA nephropathy (ACE: Angiotensin Converting Enzyme, ARB: Angiotensin Receptor Blocker, NDHP-CCB: Nondihydropyridine-type Calcium Channel Blocker,

3) Pregnant or breast-feeding patients. Patients who plan to bear children or breast-feed ,during the study and within 6 month after completion of study,

4) Hypersensitivity to the investigational drug or macrolide agents,

5) Use of potassium-sparing diuretics,

6) Persistence of liver function abnormality more than 1 month or presence of acute active hepatitis, **or**

7) Other investigational drug within last 30 days

12. Medication: Prograf 1mg capsule or Placebo capsule which is made for this study with as same shape as the tacrolimus

13. Study design

1) Study design and treatment

**0.025 mg/kg bid**

**tacrolimus or placebo**

**0.05 mg/kg bid**

**tacrolimus or placebo**

**visit 4**

**visit 5**

**visit 6**

**visit 1**

**visit 3**

**Randomization**

**16 weeks**

**0**

**4 weeks**

**8 weeks**

**12 weeks**

**visit 2**

**1 weeks**

This study was a double blind randomized controlled clinical trial and was performed in a single center (clinicaltrial.gov identifier: NCT01224028). The protocol was approved by the Institutional Review Board of Seoul National University Hospital (IRB number: H-1002-032-309) . After obtaining informed consent, we randomized patients 1:1 who had received tacrolimus to a control group (placebo) or a Tac group, in a double blind manner stratified according to whether they had taken an RAS blocker, using the computer-generated randomization lists by the independent statistical committee from the researcher (doctors, nurses, and pharmacists related to this study). After randomization, we did not change the dose or type of RAS blocker and prohibited addition of diuretics, RAS blocker, steroid, immunosuppressant, non-dihydrodipine calcium channel blocker, and omega-3 fatty acid. We followed the patients at 1 week, 4 weeks, 8 weeks, 12 weeks, and 16 weeks after the baseline visit for randomization and measured the 12-hour trough levels of tacrolimus. On each follow-up visit, except the 1-week visit, we measured clinical and biochemical parameters, including blood pressure, adverse event, compliance of medication (placebo or tacrolimus), serum creatinine, UACR, urine protein to creatinine ratio (UPCR), urinalysis, C-reactive protein, cholesterol, LDL-cholesterol, HDL-cholesterol, triglyceride, protein, albumin, liver function test, and complete blood cell count. HbA1c were measured at baseline, 8-week, and 16-week visits.

2) Treatment

The initial dose of tacrolimus was 0.1 mg/kg/day administered orally in two divided doses and was titrated to maintain trough levels at 5-10 ng/ml at each visit after randomization. The dose of tacrolimus was decided in a miligram unit. If the trough level was 10-15 ng/ml, we decreased 30% of the prescribed dosage. If the level was ≥ 15 ng/ml, we stopped tacrolimus for 2 weeks and then re-measured the trough level to adjust the dosage as described above. We withdrew the patient with the trough level of ≥ 15 ng/ml in three consecutive measurements. The level of tacrolimus was not given to the patient or the researcher but only to the statitistical committee member in charge of this study who decided on the dose of tacrolimus or placebo and notified such to the pharmacist at each visit before prescription. For adjusting the dose of placebo, the committee member should change the number of placebo capsules for the patient in the control group in the same manner as the adjustment of medication for the patient with tacrolimus who visited on the most recent day with a random allocation (no change, increase or decrease of dose, or discontinue of prescription). After 8 weeks of randomization, we reduced the dose of tacrolimus to 0.05 mg/kg/day or to half of the decided dose to maintain the trough level of 4-10 ng/ml at the 8-week visit and continued it up to 16 weeks after randomization.

3) Sample size

We estimated the sample size based on previous studies (31-35) that showed the mean percentage change of UACR 35.4% (standard deviation, 36.7%). For comparison of the control and Tac groups at a level of significance of 5% (α error = 0.05), we calculated that at least 17 patients were needed in each group to have an 80% percent power (ß error = 0.20). We allocated 20 patients to each group with an estimated ≤ 15% drop-out rate.

14. Analysis

1) Outcomes

We defined the baseline value of UACR or UPCR as the mean value of UACR or UPCR during the 3-month screening period and at visit 1 for randomization. The final level of UACR was defined as the mean value of UACR at 12-week and 16-week visits. The primary outcome was defined as the percent change (%) of the final UACR (pcUACR) compared to the baseline value [100 x (final UACR- baseline UACR)/baseline UACR]. We defined several secondary outcomes as follows; 1) the frequency of patients with percentage decrease of UACR level ≥ 30% at the 16-week visit compared to the baseline value, 2) the frequency of patients with percentage decrease of UACR level ≥ 50% at the 16-week visit compared to the baseline value, 3) the frequency of patients with UACR at the 16-week visit < 200 mg/g creatinine, 4) the pcUACR at each visit compared to the baseline visit, 5) the frequency of patients with percentage decrease of UPCR level ≥ 30% at the 16-week visit compared to the baseline value, 6) the frequency of patients with percentage decrease of UPCR level ≥ 50% at the 16-week visit compared to the baseline value, 7) the frequency of patients with UPCR at the 16-week visit < 200 mg/g creatinine, and 8) the pcUPCR at each visit compared to the baseline visit.

2) Statistical analysis

Analysis of primary and secondary outcomes was carried out by intention-to-treat (ITT). The values were expressed as mean ± standard deviation (SD) for continuous variables and the frequency for dichotomized variables. For statistical analysis, we used a paired or unpaired Student *t-*test for continuous variables. Pearson’s Chi-square test or Fisher’s exact test was used for qualitative variables. The data throughout the follow-up period were analyzed with the ANOVA for repeated measurements.

15. Measurements

|  | **visit 1** | **Visit 2** | **visit 3** | **visit 4** | **visit 5** | **visit 6** |
| --- | --- | --- | --- | --- | --- | --- |
| **Visit window** | **0 wk** | **1 wk±3d** | **4 wk±7d** | **8 wk±7d** | **12 wk±7d** | **16 wk±7d** |
| Written consent | O |  |  |  |  |  |
| Screening | O |  |  |  |  |  |
| Randomization | O |  |  |  |  |  |
| Medical exam. | O |  |  |  |  |  |
| Vital signs^1)^ | O |  | O | O | O | O |
| Height /weight^2)^ | O |  | O | O | O | O |
| CBC^3)^ | O |  | O | O | O | O |
| Chemistry^4)^ | O |  | O | O | O | O |
| Lipid panel^5)^ | O |  | O | O | O | O |
| C-reactive protein | O |  | O | O | O | O |
| Urinalysis | O |  | O | O | O | O |
| Pregnancy test | O |  |  |  |  | O |
| HbA_1c_ | O |  |  | O |  | O |
| UACR/UPCR,  Urine Na, K | O^6)^ |  | O | O | O | O |
| FK506 Trough level |  | O^7)^ | O | O | O | O |
| Medication | O |  | O | O | O |  |
| Confirm other medications | O |  | O | O | O | O |
| Compliance to medication |  |  | O | O | O | O |
| Adverse effect |  |  | O | O | O | O |
